# Optical Graphene-Based Biosensor for Nucleic Acid Detection; Influence of Graphene Functionalization and Ionic Strength

**DOI:** 10.3390/ijms19103230

**Published:** 2018-10-19

**Authors:** Diana F. Becheru, George M. Vlăsceanu, Adela Banciu, Eugeniu Vasile, Mariana Ioniţă, Jorge S. Burns

**Affiliations:** 1 Faculty of Medical Engineering, University Politehnica of Bucharest, Gh Polizu 1-7, 011061 Bucharest, Romania; diana.becheru@yahoo.com (D.F.B.); vlasceanu.georgemihail@yahoo.com (G.M.V.); adela.banciu79@gmail.com (A.B.); eugeniuvasile@yahoo.com (E.V.); jsburns@unimore.it (J.S.B.); 2 Laboratory of Cellular Therapies, Department of Medical and Surgical Sciences for Children & Adults, University Hospital of Modena and Reggio Emilia, Modena, Italy

**Keywords:** graphene, DNA, optical biosensor, fluorescence resonance energy transfer (FRET), ionic strength, quenching

## Abstract

A main challenge for optical graphene-based biosensors detecting nucleic acid is the selection of key parameters e.g. graphenic chemical structure, nanomaterial dispersion, ionic strength, and appropriate molecular interaction mechanisms. Herein we study interactions between a fluorescein-labelled DNA (FAM-DNA) probe and target single-stranded complementary DNA (cDNA) on three graphenic species, aiming to determine the most suitable platform for nucleic acid detection. Graphene oxide (GO), carboxyl graphene (GO-COOH) and reduced graphene oxide functionalized with PEGylated amino groups (rGO-PEG-NH_2_, PEG (polyethylene glycol)) were dispersed and characterized by scanning electron microscopy (SEM) and transmission electron microscopy (TEM). The influence of ionic strength on molecular interaction with DNA was examined by fluorescence resonance energy transfer (FRET) comparing fluorescence intensity and anisotropy. Results indicated an effect of graphene functionalization, dispersion and concentration-dependent quenching, with GO and GO-COOH having the highest quenching abilities for FAM-DNA. Furthermore, GO and GO-COOH quenching was accentuated by the addition of either MgCl_2_ or MgSO_4_ cations. At 10 mM MgCl_2_ or MgSO_4_, the cDNA induced a decrease in fluorescence signal that was 2.7-fold for GO, 3.4-fold for GO-COOH and 4.1-fold for rGO-PEG-NH_2_. Best results, allowing accurate target detection, were observed when selecting rGO-PEG-NH_2_, MgCl_2_ and fluorescence anisotropy as an advantageous combination suitable for nucleic acid detection and further rational design biosensor development.

## 1. Introduction

Highly informative biomarkers, in health and industry sectors, provide opportunities for cost-effective, selective and sensitive biosensing devices. For point of care diagnostic tests and quality control within industrial processes, biosensors can supersede analytical instruments. Functional nucleic acids (FNA), in particular DNA aptamers, can be conveniently synthesized and readily selected to combine very specific target discrimination with high binding affinity. FNA aptamers can be more readily manufactured than antibodies and ligands can include more elusive targets such as toxic metal ions. Effective selection and enrichment methods, plus chemical enhancement that broadens the target recognition repertoire, foster the growing popularity of FNA aptamers [1].

Detection of specific fluorescence represents a leading procedure useful for qualitative and quantitative measurement of biochemical molecules, reflecting molecular events in real time [2]. As a complementing platform, graphene-based materials have physiochemical and structural properties well-suited for electrochemical or optical biosensors [3]. A broad range of graphenic species are available for use in optical FNA detection platforms. A common aim is to exploit the graphene oxide (GO)-mediated combination of a strong broad-spectrum fluorescent quenching capacity and different adsorption affinities for FNA aptamers upon target analyte binding [4], making graphene derivatives potent nanomaterials for optical biosensor development [5]. Graphene consists of a single flat sheet of tightly packed carbon atoms arranged in a hexagonal lattice, stable under ambient conditions, bearing highly mobile and extensively-networked π electrons located above and below the graphene sheet [6].

In contrast, the prominently explored derivative GO arises through disruption of the consistent sp^2^ structure during an oxidation process via oxygen-containing addends that rearrange the carbon lattice plane, introducing defects and different hydroxyl (-OH), epoxy (-C-O-C-), carbonyl (-C=O) and carboxyl (-COOH) groups. The oxygen-based functional groups confer a hydrophilic character, making GO ideal for biomolecule attachment [7]. Modification from graphene to GO nanosheets improves miscibility with water and introduces an optical band-gap that makes GO much more photoluminescent than pure graphene [8] and useful for optoelectronics [9].

Surface modification of GO by negatively-charged carboxyl-groups to yield carboxylic acid graphene oxide (GO-COOH) can further improve aqueous dispersion and enhance biological compatibility, providing reactant sites for conjugation with positively charged peptides and proteins, plus further functionalization sites for covalent bioconjugations to the activated carboxyl group [10].

Another GO derivative, reduced graphene oxide (rGO), with partial or total oxygen depletion, has a more restored π-conjugated structure [11], rendering this nanomaterial amenable for optical biosensor development [12,13,14]. For prospective biosensors capable of direct cell measurement, rGO functionalization using polythiophene-*g*-poly (ethylene glycol) with lateral amino groups (PEG-NH_2_) has been shown to not impair the osteogenic differentiation potential of murine mesenchymal multipotent cells [15]. Functionalizing rGO with PEG-NH_2_ helped improve the solubility and stability of rGO suspensions in more complex medium solutions such as those used to support cell growth.

Fluorescence quenching by graphenic nanomaterials concerns wavelengths falling within the emission spectrum of most fluorophores. This facilitates energy transfer from an excited molecular fluorophore (the donor molecule) to another fluorophore (the acceptor molecule) via distance-dependent dipole-dipole interactions that characterise Förster resonance energy transfer (FRET) [16]. The FRET efficiency is inversely proportional (1/R^6^) to the distance (R) between the donor and acceptor, occurring over distances of 1–8 nm for GO surfaces, suitable for measuring biomolecular interactions [17] and clinical laboratory applications [18]. At distances >10 nm, photon emission by the donor molecule dominates the fluorescent signal measurement [19]. Optical detection platforms often include use of fluorescently labelled aptamer probes as donors and graphenic nanomaterials as quenchers [20].

For sensitivity and specificity, FNA biosensors rely upon the graphene-based quenching effect acting on the predefined fluorescent oligonucleotide probe changing when the specific target molecule binds in accordance with the principle of complementarity. Single-stranded DNA (ssDNA) is readily immobilized through π-π interactions between the oligonucleotide base ring structures and the GO surface. In contrast, for double-stranded DNA (dsDNA) the internalized base ring structures are hidden and outer phosphate groups evoke negative charge repulsion, leading to detachment of dsDNA from the GO surface. For such interactions, GO displayed a low photoluminescent quantum yield, predominantly functioning as an acceptor quenching the fluorescent dye [21].

Factors likely to influence biosensor performance include nanomaterial dispersion and available surface area. Ultrasonication, a cavitation process whereby micro-bubbles with super-sonic speed implosions evoke shear viscous forces that break nanomaterial sheets via mechanical stress, is a commonly preferred method for creating nanomaterial dispersions [22]. Although sonication treatment of GO preparations lacks a systematic control methodology, it was shown that exfoliation of GO can be achieved after 2 min with further sonication over 10, 30 and 60 min resulting in a reduction of the average flake size [23] proportional to the specific energy input [24,25].

Concerning electrostatic interactions between GO and oligonucleotides, the binding affinity of FNA short chains was low in comparison to long chains [26]. Unlike native graphene, GO, with abundant oxygen-containing functional groups also presents potent adsorption properties for metal cations in aqueous solution [27]. Magnesium cations, highly abundant in cellular systems, represent nature’s choice cofactor for DNA processing enzymes, serving to stabilize DNA and chromatin structure [28]. Notably, in a simple reaction system of pure water, aptamer quenching by GO had an efficiency below 30%. In contrast, in the presence of ionic salts, adsorption of shorter oligonucleotides on the graphene surface showed much faster kinetics [29] leading to a quenching efficiency close to 100%. Although dsDNA does not interact well with GO in pure water, in the presence of certain buffer salts, dsDNA can form large complexes with metal ions and remain adsorbed to GO [30]. Such findings emphasized the importance of buffer control for biosensor development.

Fluorescence signal quantification can be achieved via a direct intensity measurement or via polarization. The latter, measuring fluorescence anisotropy (FA) uses a polarization filter in front of the detector orientated either perpendicular or parallel to the incident polarized light. As a ratio between polarized components relative to total intensity, unequal intensities along the different axes of polarization result in higher values. At the molecular level, FA directly reflects the rotation of the fluorophore dipole and this in turn depends upon the fluorophore properties and microenvironment, determining the mass and size of the rotating body. Oligonucleotide aptamers are relatively small molecules that independently do not produce large FA value changes; however, nanomaterials can serve as FA amplifiers and GO is highly dispersive in water with size ranges from hundreds to thousands of nanometres. DNA interactions with GO through π-π stacking and electrostatic adsorption when forming a complex with the probe molecule allows GO to serve as both amplifier and platform to strongly alter FA values.

Numerous studies indicate that graphene-based biosensors can provide high sensitivity, ease of use and versatility, all aspects well suited for biomedical applications [31]. Many GO component factors influence biosensor performance including the method used for its synthesis and dispersion [32], the influence of pH, [33] and the effects of functionalization [34]. Such key parameters can dictate the extent of adsorption of the oligonucleotides onto the graphenic material nanosurface, either through covalent or non-covalent attachment via π-π stacking, cation-π or weak force interactions [35], ultimately establishing the sensitivity of the detection platform. 

**Figure 1:**
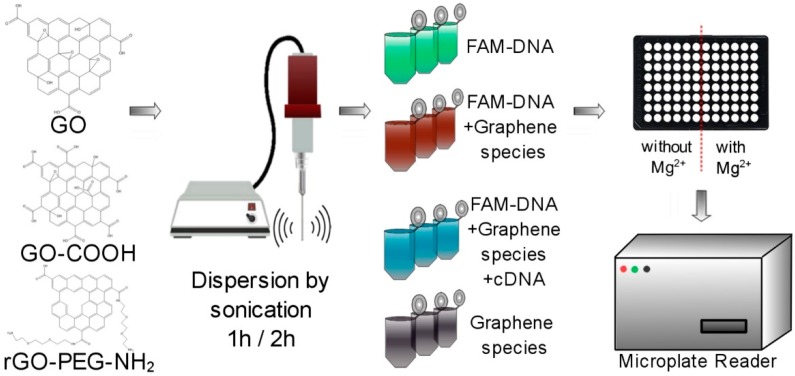
Schematic illustration depicting the experimental workflow.

**Figure 2:**
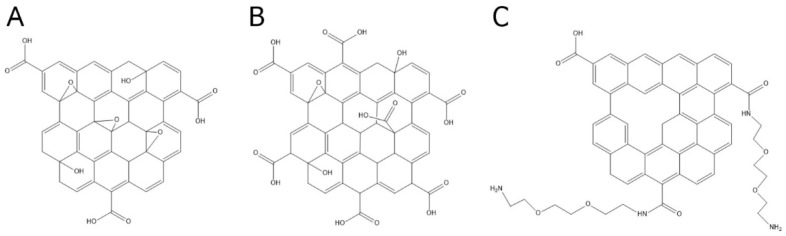
Chemical structure of graphenic species used in the assay. (**A**) Graphene oxide (GO), (**B**) carboxyl graphene oxide (GO-COOH), (**C**) reduced graphene oxide amino-PEG (polythiophene-*g*-poly) covalently linked (rGO-PEG-NH_2_).

Resorting to a simplified model of nucleic acid-GO interactions [16], we explored the quenching of a fluorescein-labelled DNA (FAM-DNA) probe and detection of the single-stranded cDNA target on three types of GO species, not previously compared directly for such purposes (Figure 1), namely; GO, GO-COOH and rGO-PEG-NH_2_ (Figure 2). Among the various conditions and graphenic species tested, we found significant differences, with rGO-PEG-NH_2_ providing more consistent results with an improved target detection performance when used with magnesium ions.

## 2. Results

### 2.1. Impact of Dispersion on Detection Platform Sensitivity

**Figure 3:**
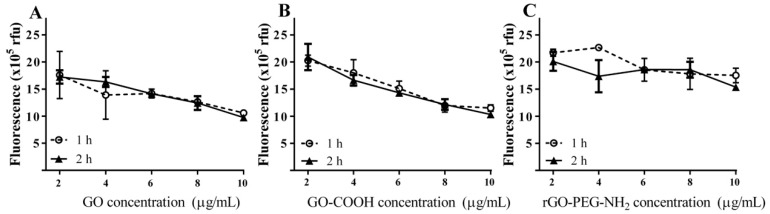
The effect of ultrasound treatment time on different species and concentrations of graphenic nanomaterial for FAM-DNA fluorescence quenching. (**A**) Graphene oxide, (**B**) carboxyl graphene oxide, (**C**) reduced graphene oxide amino-PEG covalently linked. Concentrations: graphenic materials (2–10 µg/mL), FAM-DNA probe (100 nM).

In order to benefit from their full potential, graphenic materials need to be exfoliated, typically by sonication, a procedure that could modify their topography and morphology. The influence of sonication time on the degree of graphene oxide dispersion and the potential impact on the detection platform sensitivity was investigated. GO, GO-COOH and rGO-PEG-NH_2_ were sonicated for 1 or 2 h as described in the experimental procedure. Graphene oxide samples (2, 4, 6, 8 and 10 μg/mL) were incubated with FAM-DNA probe (100 nM) for 10 min and the fluorescence measurements were performed (Figure 3).

For all the investigated graphenic species, a concentration-dependent decrease of fluorescence for both 1 h and 2 h of ultrasound treatment was recorded. At the minimum graphenic species concentration of 2 μg/mL, the sonication process did not induce significant differences. Thus, for GO and rGO-PEG-NH_2_, the FAM-DNA quenching fluorescence level decreased only slightly from 1 h to 2 h ultrasound treatment, whilst for GO-COOH the fluorescent values recorded no change. For GO, a fluorescence intensity of 1.76 × 10^6^ ± 4.3 × 10^5^ rfu was recorded for samples treated with 1 h sonication, reaching 1.72 × 10^6^ ± 1.2 × 10^5^ rfu, for samples treated for 2 h. Similarly, with rGO-PEG-NH_2_ the FAM-DNA fluorescence intensity was 2.17 × 10^6^ ± 6.5 × 10^4^ rfu for 1 h sonicated samples and 2.01 × 10^6^ ± 1.8 × 10^5^ when treated with 2 h sonication. However, at higher graphenic material concentrations the effect of the ultrasound treatment was more notable. Using 10 μg/mL nanomaterial, the quenching was accentuated, resulting in a general decrease in the fluorescence intensity. For GO, the recorded fluorescence was 1.06 × 10^6^ ± 1.9 × 10^4^ rfu after a 1 h sonication, reaching 9.74 × 10^5^ ± 5.8 × 10^3^ after 2 h sonication. For rGO-PEG-NH_2_, the FAM-DNA fluorescence values were 1.75 × 10^6^ ± 1.3 × 10^5^ rfu after 1 h and 1.54 × 10^6^ ± 1.8 × 10^4^ rfu after 2 h ultrasound treatment. As for GO-COOH, the FAM-DNA fluorescence values decreased from 1.15 × 10^6^ ± 5.9 × 10^4^, after 1 h sonication to 1.03 × 10^6^ ± 6.1 × 10^4^ rfu after 2h sonication.

In particular, rGO-PEG-NH_2_ had a relatively low quenching capacity, with significantly higher FAM-DNA fluorescence (*** *p* < 0.001) at 10 μg/mL than observed for GO or GO-COOH, for both 1 h and 2 h ultrasound treatment (Figure 3). We observed no significant differences in FAM-DNA fluorescence between GO and GO-COOH, after either 1 h and 2 h sonication treatments. In contrast, a mean difference in FAM-DNA fluorescence between GO-COOH and rGO-PEG-NH_2_ decreased from 6.02 × 10^5^ ± 6.96 × 10^4^ at 1 h sonication to 5.03 × 10^5^ ± 3.29 × 10^4^ after 2 h ultrasound treatment. 

**Figure 4:**
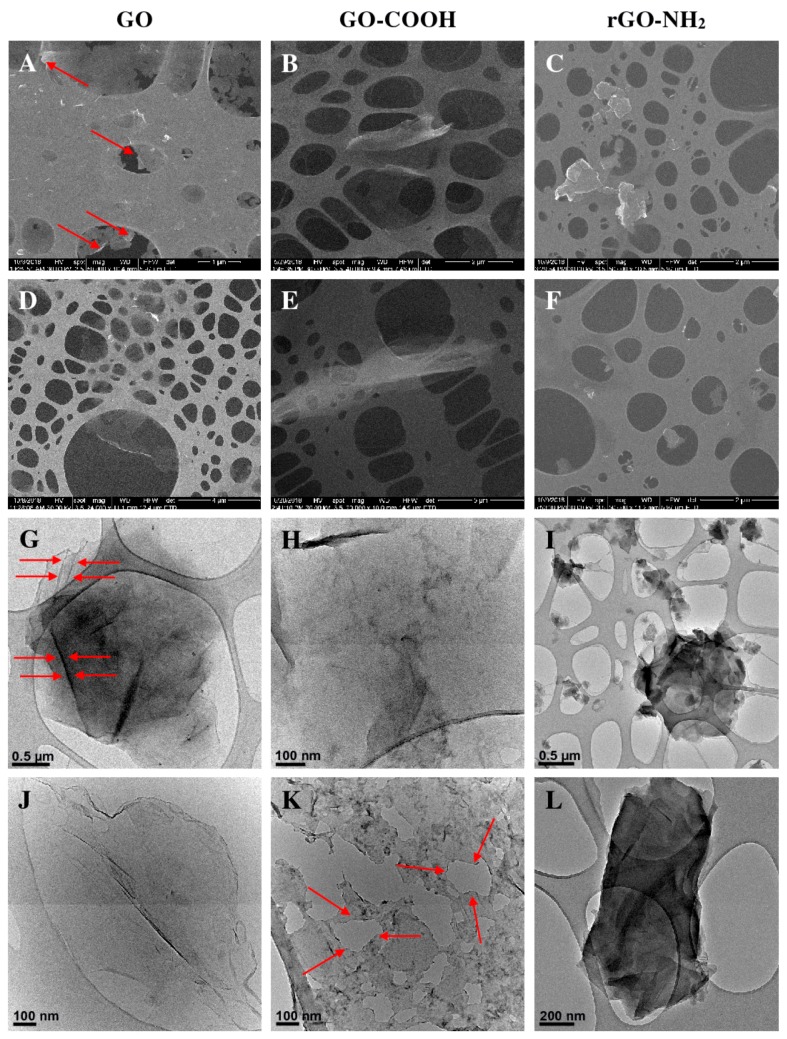
The impact of ultrasound treatment duration on GO, GO-COOH and rGO-PEG-NH_2_ as seen through scanning electron microscopy (SEM) (**A**–**F**) and TEM (**G**–**L**) images. Morpho-structural alterations induced by 1 h sonication are depicted in panel (**A**) (red arrows: incipient edge peeling) and (**G**) (red arrows: structural folding wrinkled surface) for GO, (**B**,**H**) for GO-COOH, (**C**,**I**) for and rGO-PEG-NH_2,_ whereas panels (**D**,**J**) correspond to GO, (**E**,**K**) (red arrows: layer cleavage) to GO-COOH, (**F**,**L**) to rGO-PEG-NH_2_ after 2 h sonication.

GO, GO-COOH, and rGO-PEG-NH_2_ also exhibited different morphological and structural responses to ultrasound treatment as indicated by scanning electron microscopy (SEM) and transmission electron microscopy (TEM), respectively. For GO, differences in morphology between samples treated using 1 h (Figure 4A) and 2 h sonication protocols (Figure 4D) were associated with the degree of GO flake delamination. After a 1 h sonication treatment, GO maintained some cluster conformation whereby GO flakes were moderately exfoliated and featured peeling around the edges (Figure 4A). As sonication increased, the GO agglomeration size narrowed and its thickness diminished to fewer layers. This observation was confirmed by TEM, GO was reasonably dispersed and exfoliated after only a 1 h ultrasound treatment; furthermore, key features of graphene oxide architecture were preserved: a specific structural folding wrinkled surface (Figure 4G) and precise separation of the GO layers.

The SEM image of GO-COOH sonicated for 1 h (Figure 4B) also illustrated the typical morphology of graphene—a bendable multilayer flake. Similar folding behaviour was observed after 2 h sonication treatment (Figure 4E). However, greater stack transparency indicated a reduced thickness, thus a higher dispersion of the carbon nanomaterial. Although at the microscale the only apparent changes indicated graphenic thinning, at the nanoscale, the carbon layer alterations were more profound (layer fragmentation). After the 1 h sonication time (Figure 4H) the GO-COOH flakes of various thicknesses were uniformly spread onto the surface, in contrast, after doubling the sonication period, the exfoliated layers were thinner and presented large cleavages (Figure 4K).

Unlike GO-COOH, rGO-PEG-NH_2_ showed a distinct behaviour. The SEM images revealed more desirable dispersion after 2 h sonication (Figure 4F) compared to the 1 h treated samples (Figure 4C). TEM showed that graphenic agglomeration decreased in area, yet preserved its thickness. Hence, rGO-PEG-NH_2_ sheet interactions were stronger in comparison to the other graphenic species. The thick ensembles resisted the sonication procedure due to the network of PEG-amine chains grafted along the edges of the reduced graphene sheets. The manner in which rGO-PEG-NH_2_ aggregated was split during sonication (Figure 4I,L) and was explained by disintegration occurring throughout the aggregated layers rather than by inter-layer force disruption. Since the 2 h sonication treatment ensured better dispersion of the graphenic materials, providing more homogeneous stable solutions, this protocol was chosen for further experiments.

### 2.2. Interaction between DNA and Graphene Platforms in Aqueous Solution

**Figure 5:**
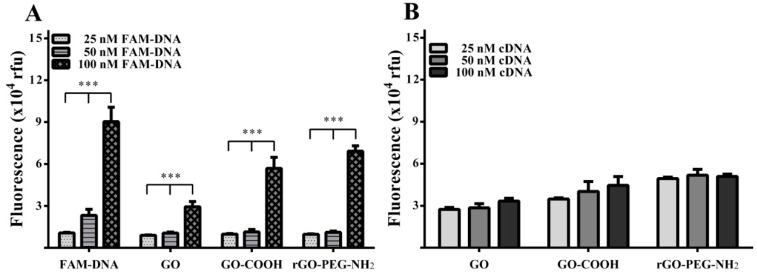
Fluorescence values for different FAM-DNA probe concentrations and three diverse graphenic nanomaterials (10 μg/mL). (**A**) Fluorescence intensity of different increasing FAM-DNA probe concentrations in the presence of distilled water, GO, GO-COOH, and rGO-PEG-NH_2_. (**B**) Fluorescence of FAM-DNA probe (100 nM) and graphenic material (10 μg/mL) with addition of increasing concentrations of target cDNA. Error bars represent means ± standard deviation (SD) of *n* = 3. *** *p* < 0.001.

Analysis of specific interactions between the three graphenic species and FAM-DNA was first performed in aqueous solution using FAM-DNA probe concentrations ranging from 25 to 100 nM, keeping graphenic material concentrations constant (10 μg/mL). For lower concentrations of FAM-DNA probe (25 and 50 nM), the fluorescence was not significantly altered, reaching a steady-state level of approximately 1 × 10^4^ for 50 nM FAM-DNA probe (Figure 5A). When using 100 nM FAM-DNA probe a significantly higher fluorescence intensity value was obtained for all graphenic nanomaterials (*** *p* < 0.001), reflecting saturation of the graphenic fluorescence quenching effect. In comparison to the 50 nM FAM-DNA values, there was a ≈3-fold increase for GO, a ≈5-fold increase for GO-COOH and ≈6-fold for rGO-PEG-NH_2_. To further evaluate the interaction between FNA and graphenic species, we measured the fluorescence intensity when FAM-DNA (100 nM) was introduced to varying concentrations of single-stranded cDNA (25, 50, 100 nM) in the presence of graphenic material (10 µg/mL). Although using GO and GO-COOH, we observed a small incremental trend for fluorescence intensity, this was not the case for rGO-PEG-NH_2_, where there was no apparent target cDNA concentration dependent release of FAM-DNA from quenching (Figure 5B).

### 2.3. Assessment of the Effects of Magnesium Ions on Fluorescence Intensity

**Figure 6:**
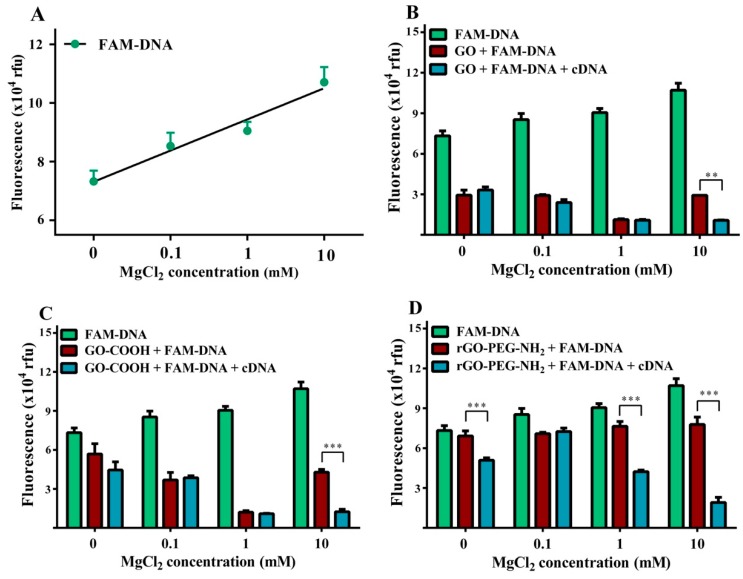
Representation of fluorescence values in the presence of 0.1 mM, 1mM and 10 mM MgCl_2_ for (**A**) 100 nM FAM DNA, coefficient of determination R^2^ = 0.903, *p* < 0.0001. (**B**). Graphene oxide, (**C**) carboxyl graphene oxide, (**D**) reduced graphene oxide amino-PEG covalently linked. Concentrations: 10 μg/mL graphenic materials, 100 nM FAM-DNA and 100 nM complementary DNA. Values represent mean ± SD of *n* = 3. (** *p* < 0.01, *** *p* < 0.001).

Three different magnesium chloride concentrations (0.1, 1 and 10 mM) were tested to determine the impact of magnesium chloride addition on detection platform sensitivity. In the absence of a graphenic substrate, addition of MgCl_2_ caused a significant concentration-dependent increase in the FAM-DNA fluorescence intensity (Figure 6A) up to ≈1.5-fold greater (*p* < 0.001) at the 10 mM concentration. There was a graphenic species specific effect, GO and GO-COOH behaving differently from GO-PEG-NH_2_. The former two graphenic species induced a significant concentration-dependent FAM-DNA quenching effect, that reduced fluorescence intensity by a maximal 8-fold when using 1 mM MgCl_2_, but under these conditions using rGO-PEG-NH_2_, the FAM-DNA fluorescence intensity dropped only 1.2-fold. Notably, the single stranded cDNA target molecule only induced a significant change in fluorescence intensity when using 10 mM MgCl_2_, (** *p* < 0.01 for GO, Figure 6B and *** *p* < 0.001 for GO-COOH, Figure 6C). In contrast, rGO-PEG-NH_2_ showed a consistently low FAM-DNA quenching effect for all MgCl_2_ concentrations (Figure 6D). The addition of target cDNA more reproducibly reduced the fluorescence intensity; already ≈1.7-fold at 0.1 mM MgCl_2_, and ≈3.8-fold with 10 mM MgCl_2_. Thus rGO-PEG-NH_2_ achieved significant (*** *p* < 0.001) target cDNA detection ability at a lower (1 mM) MgCl_2_ concentration than observed for the other two graphenic species.

**Figure 7:**
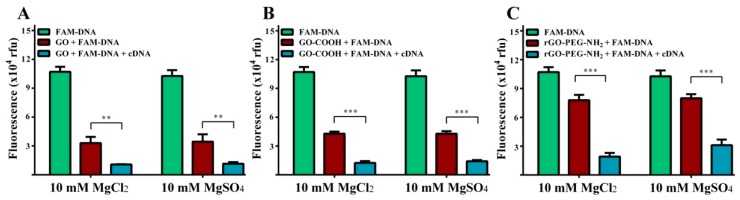
Representation of fluorescence values in the presence of 10 mM MgCl_2_ and 10 mM MgSO_4_, for different graphenic species (**A**) GO, (**B**) GO-COOH and (**C**) rGO-PEG-NH_2_ and 100 nM FAM-DNA plus100 nM cDNA. Values represent mean ± SD of *n* = 3. (** *p* < 0.01, *** *p* < 0.001).

Anions of different ionic charge states, Cl^−^ and SO^4−^, were compared in order to verify whether the Mg^2+^ metallic ion was principally responsible above characteristic fluorescence level changes (Figure 7). Testing a 10 mM FAM-DNA probe concentration, there was very close fluorescence intensity agreement between MgCl_2_ and MgSO_4_ with near-equivalent corresponding values for all conditions tested for each graphenic species. The presence of the target cDNA induced a signal decrease resulting in significant differences for GO (** *p* < 0.01), GO-COOH and rGO-PEG-NH_2_ (*** *p* < 0.001), regardless of the salt anion. Nonetheless, using rGO-PEG-NH_2_, target cDNA addition induced a more pronounced modification of the fluorescent level in the presence of 10 mM MgCl_2_ than 10 mM MgSO_4_ (*p* < 0.05).

### 2.4. Fluorescence Anisotropy 

**Figure 8:**
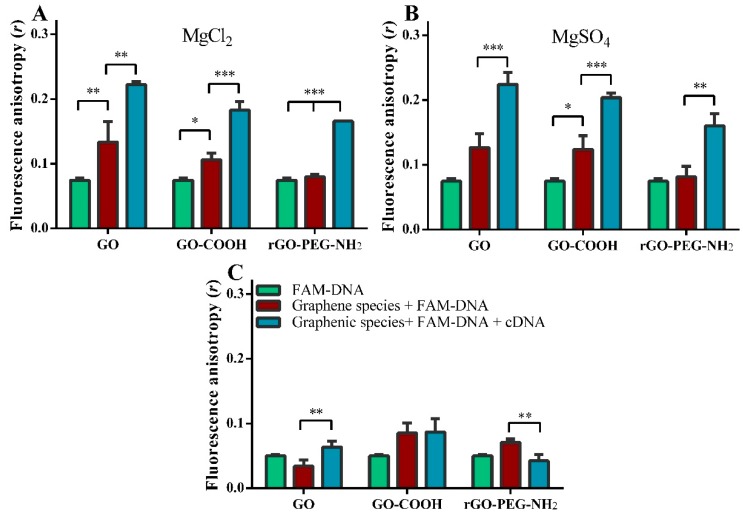
Fluorescence anisotropy in the presence of (**A**) MgCl_2_, (**B**) MgSO_4_ and (**C**) the absence of magnesium ions. Concentrations: 100 nM FAM-DNA, 100 nM cDNA, 10 μg/mL graphene oxide materials, 10 mM MgCl_2_ and 10 mM MgSO_4_. (* *p* < 0.05, ** *p* < 0.01, *** *p* < 0.001).

Fluorescence anisotropy was measured with or without both metallic salts (Figure 8). FA values in either MgCl_2_ or MgSO_4_ were very similar. Significant increases in fluorescence anisotropy in the presence of Mg^2+^ were obtained when target cDNA was added to FAM-DNA for each graphenic species; FA increased 1.7-fold (** *p* < 0.01), 1.8-fold (*** *p* < 0.001) for GO-COOH and 2-fold (*** *p* < 0.001) rGO-PEG-NH_2_, (Figure 8A,B).

In contrast, under metallic salt-free aqueous conditions, all FA values were lower and cDNA did not change the fluorescence anisotropy value of FAM-DNA for GO-COOH. For GO, target cDNA doubled the FA reading (** *p* < 0.01). Uniquely for rGO-PEG-NH_2_, the addition of 100 nM target cDNA to the 100 nM FAM-DNA probe induced a drop in FA value from 0.07 ± 0.01 to 0.04 ± 0.01 (** *p* < 0.01) (Figure 8C).

## 3. Discussion

In the present study, key conditions including graphenic material dispersion, functionalization and ionic strength, each capable of influencing the sensitivity of optical FNA biosensor platforms were investigated by measuring fluorescence intensity and polarized fluorescence during probe–target interactions on diverse graphenic species. Various concentrations of FAM-DNA probe and single-stranded cDNA target were compared and interaction between FAM-DNA and graphenic species was assessed by fluorescence anisotropy.

### 3.1. Microstructural Characterization and Degree of Dispersion

Ultrasound treatment was performed as described [36] aiming to ensure an adequate dispersion of the graphenic material in suspension, thus facilitating reactions between the nanomaterial and FNA. The extent of nanoparticle dispersion in relation to sonication treatment time was analysed by SEM, TEM and indirectly by measuring the fluorescence quenching performance of increased concentrations of graphenic nanomaterial.

Complementary SEM (Figure 4 A–F) and TEM (Figure 4 G–L), investigation methods showed that the extent of sonication treatment diversely altered the morpho-structural features of the graphenic sheets. Prolonged sonication procedures enhanced the dispersion of GO. GO-COOH or rGO-PEG-NH_2_ with higher degrees of homogeneity, improved sheet exfoliation and/or fragmentation. Therefore, a 2 h sonication treatment protocol was selected for further assays regarding the graphene-based platform cDNA target detection.

The impact of ultrasound waves was similar for GO and GO-COOH while different behaviour was observed for rGO-PEG-NH_2_. Dissimilarities were attributed to the specific graphene functionalities that affected the physical stability of the layers. Generally, the degree of delamination increased with sonication time; however, the mechanisms differed. For GO, the sonication-improved exfoliation was not accompanied by obvious defect formation in the monolayers. On the other hand, prolonged sonication caused discontinuities and clear disruptions in the GO-COOH layers. Distinguishing behaviour was observed for rGO-PEG-NH_2_ which was less prone to delaminate and more susceptible to break into smaller but compact aggregates. This increased stability of rGO-PEG-NH_2_ to mechanical stimuli could be justified by the relative lack of defects and functionalities of the reduced graphene layer, that could strengthen the carbon sheet framework. Nonetheless, common features of 2D carbon nanomaterials, such as wrinkles and overall irregularity persisted for the three materials, so they could continue to support the adsorption of ssDNA [37,38].

Of potential influence also, was a lower degree of dispersion of rGO-PEG-NH_2_ indicated by SEM and TEM. Poorer graphenic nanomaterial dispersion would reduce the surface area available for FAM-DNA probe adsorption and fluorescence quenching. Despite evidence of specific structural or morphological alterations according to the sonication protocol used, the influence of different sonication times on the fluorescence quenching performance was not significant. This likely reflected our conservative sonication time choice for comparison. Earlier studies showed that for sonication treatments shorter than 30 min, GO aggregates persisted and disrupted dispersion homogeneity [39]. Conversely, under prolonged ultrasound exposure, excessive deterioration accompanied the delamination of the graphene flakes into graphene sheets [40,41].

### 3.2. Mechanisms of Interaction between DNA and Graphenic Platforms

Use of a 100 nM FAM-DNA concentration was helpful to ensure that the graphenic platform was saturated with aptamer probe. By further addition of complementary target cDNA, a slight increase in fluorescence intensity for GO and GO-COOH was observed, while for rGO-PEG-NH_2_ fluorescence intensity values were unchanged. These results highlighted the existence of different specific interaction mechanisms between different graphenic species and both FAM-DNA and cDNA, extending similar studies restricted to GO [42,43,44]. The traditional concept regarding interaction between DNA and graphenic platforms, useful for biosensor design, proposed a preferential probe attachment via DNA homopolymer binding on the nanomaterial, with an adsorption energy difference between probe and target [42]. This requisite was met by the observation that dsDNA interacted with GO with lower affinity than ssDNA [45]. Later, Paul et al., confirmed that addition of an oligonucleotide target molecule with complementary sequence to the ssDNA probe activated an in situ hybridization that induced desorption of the dsDNA probe/target complex from the GO surface [20]. The electrostatic interaction established between dsDNA and GO was weaker than the effect of hydrophobicity. Independent studies have proposed alternative possible molecular interaction mechanisms between fluorophore labelled-ssDNA probe, complementary target cDNA and graphene oxide [42,43,45]. According to a Langmuir–Hinshelwood mechanism, the target cDNA is first adsorbed on the GO surface and then makes a duplex with the preadsorbed ssDNA probe before they co-detach from the substrate. An alternative Elay–Rideal mechanism involves direct binding of the cDNA to the pre-adsorbed probe via DNA hybridization [43].

Although our study did not explore these alternatives in detail, we were initially disappointed by only very modest increases in fluorescence intensity upon incremental addition of increased concentrations of target cDNA. However, critical of the Langmuir–Hinshelwood hypothesis, Liu et al. highlighted that cDNA target signal generation from an adsorbed DNA-FAM probe was not likely to be efficient and the target cDNA could be assumed to be divided into three populations. The majority staying in solution phase, a fraction existing as dsDNA paired with desorbed DNA-FAM and the remaining single-stranded cDNA adsorbed by the GO surface to displace the DNA-FAM probe. So, each target cDNA need not generate one FAM-DNA hybridization event leading to one desorbed fluorophore. Instead, more consistent with our observations, their data suggested that with 200 nM added cDNA only a 1.5–4% equivalent of the probe FAM-DNA would be desorbed [42]. It is also noteworthy that Huang and Liu failed to see probe desorption from GO when the target cDNA sample was dispersed in water [17].

Notably, rGO-PEG-NH_2_ displayed a weaker FAM-DNA fluorescence-quenching capacity than either GO or GO-COOH. This result may be attributed to the presence of the rGO surface covalently linked diamino PEG chains that at neutral pH are protonated, while for GO and GO-COOH the carboxyl groups are contrastingly deprotonated [29]. A likely interaction mechanism to explain this result would be that positively charged NH^3+^ groups from the PEG chain caused repulsion of the exposed positively charged bases of the single stranded FAM-DNA probe. This need not discount the possibility that other aspects of the PEG-NH_2_ functionalization help prevent close FAM-DNA interaction with the rGO surface, thus impairing fluorescence quenching. Conversely, the result may also reflect a lower degree of dispersion of rGO-PEG-NH_2_ as indicated by SEM and TEM. Poorer graphenic nanomaterial dispersion would reduce the surface area available for FAM-DNA probe adsorption and fluorescence quenching.

Regarding rGO-PEG-NH_2_, Zhang et al., observed that PEG could reduce the rate of DNA adsorption to GO, yet also enhance cDNA hybridization kinetics to a surface-linked FAM-DNA probe [46]. A weaker DNA surface adsorption would be consistent with our data, and enhanced cDNA hybridization kinetics for rGO-PEG-NH_2_ would favour an Elan–Rideal mechanism more consistent with our anisotropy data, indicating that in contrast to GO and GO-COOH results, FAM-DNA interactions with rGO-PEG-NH_2_ were more restricted to the cDNA complexed form. 

### 3.3. A Beneficial Influence of Magnesium

A number of synthetic fluorescent dyes alter their fluorescent properties upon binding Mg^2+^ and we observed that this applied to our 6-FAM labelled DNA probe also. In the presence of graphenic species, a Mg^2+^ concentration-dependent FAM-DNA quenching effect observed for both GO and GO-COOH was not the case for the third graphenic species. Instead, rGO-PEG-NH_2_ showed a reduced, more consistent quenching effect, unaltered by increasing concentrations of MgCl_2_ or MgSO_4_. Furthermore, rGO-PEG-NH_2_ did show a Mg^2+^ concentration-dependent degree of quenching upon addition of the cDNA target with twice as much quenching with 10 mM rather than 1 mM MgCl_2_. Overall, results in the presence of magnesium ions were more reproducible, consistent with the observations of Lei et al., that metallic ions, such as Mg^2+^, Ca^2+^, Na^+^ and K^+^ induced a good distribution of DNA macromolecules on the surface of graphene oxide and facilitated the formation of dsDNA/GO and/or ssDNA/GO complexes as metal ions neutralized the electrostatic repulsions between the macromolecule and the nanomaterials [30]. Moreover, our results confirmed that the presence of metallic ions facilitated the quenching of the dsDNA on GO and GO-COOH surfaces [47]. Double-helix formation induces a conformational length change of the joining single-stranded molecules. Magnesium ions decrease the flexibility of the dsDNA whilst screening the external negative charge of the phosphate backbone [48], allowing the dsDNA to avoid charge repulsion, bringing it into the proximity of rGO [49]. Highly reproduced consistent results, despite the use of very different magnesium salt anions, confirmed that the Mg^2+^ component was chiefly responsible for an overall interaction assay enhancement, encouraging its recommendation for optical detection platforms that rely on graphenic nanomaterial-quenching effects.

### 3.4. Fluorescence Anisotropy

Fluorescence anisotropy has been used extensively to detect different molecules, ions, cells and for studying their interactions [50]. Small molecules unable to conserve their widely dispersed energy, such as fluorophore-linked aptamers like FAM-DNA, generate a low signal. However, when they bind to larger molecules, stabilization can conserve the emitted light, thus the mobility decreases and the polarized fluorescence anisotropy signal increases. For our purposes, in the presence of a target cDNA molecule, the adsorbed FAM-DNA probe detaches from the relatively large graphenic surface, resulting in an increased mobility and decreased anisotropy [51]. In experiments performed in aqueous conditions rGO-PEG-NH_2_ showed a decreased anisotropy, in the presence of the target cDNA molecule, implying a specific target-mediated detachment of hybridized FAM-DNA/cDNA from the surface of rGO-PEG-NH_2_. Conversely, for GO, the presence of target cDNA caused an increase in anisotropy, most likely indicating that most of the hybridized FAM-DNA/cDNA detached poorly, remaining adsorbed to the GO. One may assume a direct binding of cDNA to FAM-DNA on the GO surface was found to inhibit rather than promote DNA hybridization on its surface [50], and the increased anisotropy signal does not necessarily discriminate and exclude the possibility that crowding from non-specifically adsorbed cDNA indirectly constrained the freedom of movement the FAM-DNA.

On the other hand, in MgCl_2_ and MgSO_4_ solutions, FAM-DNA fluorescence anisotropy increased in the presence of target DNA in a material-independent manner, demonstrating the dominant influence of Mg^2+^ ions to prevent the FAM-DNA probe desorption from the graphenic substrate, as shown by others [17]. Most relevant to biosensor specificity, we observed that the FAM-DNA anisotropy measurement was not influenced by rGO-PEG-NH_2_ alone; for this graphenic species in particular, a change in fluorescence anisotropy required the presence of the target cDNA.

## 4. Materials and Methods 

Graphene oxide (GO) and magnesium chloride ≥98.0% (MgCl_2_) were purchased from Sigma-Aldrich (St. Louis, MO, USA). Carboxyl graphene oxide (GO-COOH) and aminated graphene amino-PEG covalently linked (rGO-PEG-NH_2_) were purchased from ACS Material (Pasadena, CA, USA). Magnesium sulphate ≥99.0% was acquired from Fluka (USA). With reference to a study of nucleic acid-GO interactions (Zhou et al., 2013), the T7 RNA polymerase promoter sequence DNA aptamer probe sequence was supplied 3’-labelled with 6-fluorescein amidite (6-FAM), according to the sequence 5′-TTT CAA CAT CAG TCT GAT AAG CTA TCT CCC-3′/6-FAM (referred to as FAM-DNA). The corresponding single-stranded DNA target molecule with complementary sequence 5′-GGG AGA TAG CTT ATC AGA CTG ATG TTG AAA-3′ (cDNA) was purchased from Integrated DNA Technologies, Inc (Coralville, IA, USA). 

### 4.1. Preparation of Graphenic Nanomaterials Samples

Aqueous dispersions of graphenic materials were prepared at an initial concentration of 1 mg/mL. All samples were dispersed via ultrasound treatment conducted for either 1 or 2 h, on an ice bath, using a VC × 750 sonication probe for small and medium volumes (Sonics & Materials, Inc, Newtown, CT, USA), operated with a 750 W source, at a frequency of 20 kHz. A 10 second (s) pulse, 5 s pause was adopted, i.e., during a 1-h treatment the graphenic materials experienced 40 min of sonication. Subsequently, the graphenic materials were serially diluted in water at concentrations ranging between 2 to 10 µg/mL and re-dispersed for 5 or 10 min, using a consistent sonication protocol, before each assay. For the morpho-structural investigations of GO, GO-COOH and rGO-PEG-NH_2_ further dilution of the 1 mg/mL graphene dispersions to a concentration of 1 µg/mL were performed; 50 µL of the diluted dispersion were dropcasted onto a carbon-coated copper TEM grid support (Christine Gröpl, Tulln, Germany) and left undisturbed for solvent evaporation. After drying, duplicate independent samples were characterized by SEM and TEM, recording at least 15 microscopy images of each sample to obtain representative figures.

### 4.2. Graphene Oxide (GO)-Based Biosensor Preparation

Fluorescence evaluation was conducted by transferring the graphenic samples individually into triplicate wells of a black 96-well microplate (Costar, Corning Inc, New York, NY, USA), with distilled water as control. DNA samples concentration and purity were evaluated using Denovix DS-11+ ultraviolet-visible (UV-Vis) spectrophotometer (DeNovix, Wilmington, DE USA). FAM-DNA at varied concentrations; 25 nM, 50 nM or 100 nM was added, incubating for 10 min at room temperature to establish a molecular interaction equilibrium before measurement. Subsequently, variable concentration of analyte target ssDNA, 25 nM, 50 nM or 100 nM, were added to triplicate wells and the solutions were left for 10 min at room temperature before measurement.

### 4.3. Magnesium Ions Effect

Different concentrations of MgCl_2_ (0.1 mM, 1 mM or 10 mM) or MgSO_4_ (10 mM) were added, before subsequent addition of FAM-DNA probe to 10 μg/mL graphenic samples, up to a final volume of 100 μL in order to study the impact of a magnesium salt environment on interactions between DNA and graphene materials. 

### 4.4. Fluorescence and Microscopy Analysis

Experiments were conducted using an Anthos Zenyth™ 3100 fluorescence microplate reader, (Beckman Coulter, Brea, CA, USA), with excitation filter 485 nm and emission filter 535 nm. Fluorescence values were measured for 5 min and 35 s, at 29.5 °C, using a polarized fluorescence method, based on the Förster resonance energy transfer (FRET) technique. All results were measured simultaneously, in triplicate. 

The morpho-structural investigations of GO, GO-COOH and rGO-PEG-NH_2_ sheets were performed by using the scanning (SEM-QUANTA INSPECT F) and transmission (TEM-TECNAI F30 G2STWIN) electron microscopes.

### 4.5. Data Statistical Analysis

Raw data was presented using mean and standard deviation. To determine the statistical significance, a one-way analysis of variance (ANOVA) analysis was performed and a post-hoc Tukey test was applied for determining statistical significance.

Fluorescence anisotropy (FA) was calculated as follows:

(1) $$r=\frac{I\;\|-G*I⊥}{I\;\|+2G*I⊥}$$

where *r* represents the emission of anisotropy, *G* is the correction factor of the device whilst *I*‖ and *I*⊥ represent the fluorescence intensity.

## 5. Conclusions

The study aimed to evaluate specific graphenic nanomaterial species, under various experimental conditions, to establish a sensitive DNA aptamer-based FNA detection platform. We highlighted the main parameters that influenced the detection of a complementary ssDNA target molecule, revealing only a modest influence of different ultrasound treatments and greater significance of the graphenic species and inclusion of magnesium ions, when testing different concentrations of fluorophore-labelled probe and target DNA.

Ultrasound treatment achieved effective delamination of all studied graphenic species sheets. SEM and TEM analysis showed that the extent of delamination was comparable for GO and GO-COOH and slightly lower for rGO-PEG-NH_2_. Layer defects associated with exfoliation treatment were observed for GO-COOH. The 2 h ultrasound treatment achieved a degree of dispersion for the graphenic materials appropriate for application in a nucleic acid detection platform.

Regarding the interaction between the single-stranded FAM-DNA probe, single-stranded cDNA target and graphenic species, the results in aqueous solution were far less consistent that those obtained when using magnesium ions. The fluorescence intensity measurement of the control FAM-DNA probe increased in a Mg^2+^ concentration-dependent manner and magnesium ions served to improve the consistency of molecular interactions. With addition of graphenic nanomaterial, the FAM-DNA fluorescence intensity was inversely proportional to the corresponding fluorescence anisotropy with significant differences between the graphenic species. Experiments conducted in either aqueous or Mg^2+^ containing conditions, revealed differences between the three graphenic species. The tendency for FAM-DNA quenching was similar in both contexts, but only significant when using Mg^2+^, the order of preference for FAM-DNA probe quenching being GO > GO-COOH > GO-PEG-NH_2_.

Most significantly, regarding the interaction between the target cDNA and graphenic material two possible mechanisms emerged, the direct binding of cDNA to FAM-DNA in the case of rGO-PEG-NH_2_ and a more indirect competitive mechanism for GO and GO-COOH confirmed by fluorescence measurements and fluorescence anisotropy results.

We established a prototype “turn-off” biosensor, that functions in an accurate manner to detect target cDNA. The enhanced specificity and sensitivity from the combination of rGO-PEG-NH_2_, magnesium ions and fluorescence anisotropy provided an advantageous platform for the further development of graphene-based optical biosensors for nucleic acid detection.
